# X Chromosome Inactivation and Differentiation Occur Readily in ES Cells Doubly-Deficient for MacroH2A1 and MacroH2A2

**DOI:** 10.1371/journal.pone.0021512

**Published:** 2011-06-30

**Authors:** Borko Tanasijevic, Theodore P. Rasmussen

**Affiliations:** 1 Center for Regenerative Biology, University of Connecticut, Storrs, Connecticut, United States of America; 2 Department of Molecular and Cell Biology, University of Connecticut, Storrs, Connecticut, United States of America; 3 Department of Pharmaceutical Sciences, University of Connecticut, Storrs, Connecticut, United States of America; Florida State University, United States of America

## Abstract

Macrohistones (mH2As) are unusual histone variants found exclusively in vertebrate chromatin. In mice, the *H2afy* gene encodes two splice variants, mH2A1.1 and mH2A1.2 and a second gene, *H2afy2,* encodes an additional mH2A2 protein. Both mH2A isoforms have been found enriched on the inactive X chromosome (Xi) in differentiated mammalian female cells, and are incorporated into the chromatin of developmentally-regulated genes. To investigate the functional significance of mH2A isoforms for X chromosome inactivation (XCI), we produced male and female embryonic stem cell (ESC) lines with stably-integrated shRNA constructs that simultaneously target both mH2A1 and mH2A2. Surprisingly, we find that female ESCs deficient for both mH2A1 and mH2A2 readily execute and maintain XCI upon differentiation. Furthermore, male and female mH2A-deficient ESCs proliferate normally under pluripotency culture conditions, and respond to several standard differentiation procedures efficiently. Our results show that XCI can readily proceed with substantially reduced total mH2A content.

## Introduction

The most extreme epigenetic modification that occurs on the nucleosome level is the substitution of core histones with non-canonical variants. Macrohistones (mH2As) are non-allelic variants of the conventional histone H2A and are defined by the presence of a large (∼30 kDa) C-terminal non-histone domain connected to the H2A-like domain through a short linker [1]. Thus, mH2As are nearly 3 times the molecular weight of canonical H2A histones. The mouse genome contains two genes, *H2afy* and *H2afy2,* that encode separate proteins called macroH2A1 and macroH2A2 (abbreviated mH2A1 and mH2A2) [2], [3]. In addition, the mRNA product of *H2afy* is subject to alternative splicing to produce two distinct protein isoforms, mH2A1.1 and mH2A1.2 that differ in the non-histone region [4]. The two genes map to different chromosomes in both mice and humans, exhibit highly similar exon structures, and encode protein products with a high degree of amino acid identity [2], [3]. In addition, the mouse genome databases indicate the existence of a third macrohistone gene (termed *H2afy3*), but this locus is most likely a processed pseudogene that does not encode protein [5].

A number of prominent studies of mH2As have focused on their potential role in X chromosome inactivation (XCI), and cytological studies have identified concentrated mH2A1 localization to the inactive X chromosome (Xi), which can be detected by immunofluorescence as a macrochromatin body (MCB) [6]. Additionally, mH2A2 has been found enriched on the single Xi in mammalian female diploid cells [2], [3]. Sensitive assays show an approximately 1.5-fold enrichment of mH2A1 on the Xi compared to the autosomes [7]. Deletion of Xist, a nuclear RNA required for XCI that associates exclusively with Xi, causes MCBs to become undetectable in differentiated cells [8]. However, ectopic expression of Xist RNA on autosomes is sufficient to initiate the formation of MCBs [9]. MCB formation represents a relatively late epigenetic event during random (somatic) XCI, suggesting a potential role for mH2As in the maintenance of large heterochromatic genomic regions [9], [10]. On the other hand, imprinted XCI that occurs in the cells of the trophoblast lineage is characterized by mH2A1 deposition during early stages of inactivation, indicating a possible role for macrohistones in the initiation of transcriptional silencing of the paternal X chromosome [11]. Reactivation of the Xi has been observed upon depletion of mH2A1, but only in the presence of inhibitors of DNA methylation and histone deacetylation [12]. In addition to Xi, mH2A1 associates with other types of silent chromatin to include sex vesicles (XY-bodies) that form during male gametogenesis [13] and senescence-associated heterochromatic foci (SAHF) in post-mitotic cells [14]. Furthermore, direct evidence for the involvement of mH2A1 in the repression of individual gene loci has also been demonstrated [15], [16], [17].

The repressive mode of action for the macrohistones has been attributed to the interference with p300-dependent histone acetylation and the hindrance of transcription factor NF-κβ binding, as well as the inhibition of nucleosome remodeling and repositioning by SWI/SNF and ACF [18], [19]. The later finding has recently been challenged by data showing that mH2A1-containing nucleosomes were efficiently mobilized by both complexes, although mH2A1 specifically reduced SWI/SNF recruitment to a DNA template containing a nucleosome positioning sequence [20]. In agreement with the proposed repressive function, specific deposition of mH2A1 into the inactive allele of a subset of imprinted genes has been observed [21]. In contrast, transcribed regions of active genes, including genes that escape X inactivation, were significantly devoid of this histone variant [22], [23]. However, a recent report indicates that a distinct subset of expressed genes contain mH2A1 in transcribed regions, implying a novel cellular function for macrohistones for the protection of genes from silencing [24]. In addition, both mH2A1 and mH2A2 have been implicated in the regulation of developmentally important genes [25].

Based on the above evidence, we hypothesized that macrohistones might be important for the initiation and maintenance of XCI. However, the presence of two macroH2A encoding genes, alternative splicing, and a related (though likely untranslated) pseudogene complicates the analysis through genetic approaches. We therefore devised a strategy to create ESCs that are doubly-deficient for mH2A1 and mH2A2 through the use of stable shRNA constructs that produce interfering RNAs that target mH2A1 and mH2A2 mRNAs. Here we present the surprising result that combined deficiency for mH2A1 and mH2A2 has no major effect on the establishment and maintenance of XCI. In addition, several established procedures for the differentiation of ESCs are largely unaffected by mH2A1/mH2A2 deficiency. Our results suggest that studies that rely heavily on the simple association of mH2As with chromatin (rather than genetic analysis) should be carefully evaluated since physical association does not necessarily equate with functional significance.

## Results

### Stable double shRNA knock down of macroH2A isoforms and splice forms

In mice, similar macroH2A proteins are expressed from two genes, *H2afy* and *H2afy2* (Figure S1A), and alternative splicing of H2afy transcripts produces two proteins [2], [3], [4]. In total, at least three mH2A protein isoforms can be co-expressed in the same cell. The situation is further complicated by the existence of an expressed pseudogene from a third gene *H2afy3*, though this gene is not thought to encode a protein. We were interested in conducting a genetic analysis to determine the functional significance of mH2As in ESCs. In order to undertake such an analysis, we first needed to develop robust PCR-based assays that could distinguish between splice forms and transcripts emanating from *H2afy*, *H2afy2*, and *H2afy3*. Transcripts from *H2afy* produce mRNAs encoding splice forms mH2A1.1 and mH2A1.2. These can be readily and unambiguously detected by using primers anchored in alternatively spliced exons. However, *H2afy2* and *H2afy3* are quite similar to one another at the level of expressed RNA. We utilized the presence of several expressed sequence variations that differ between *H2afy2* and *H2afy3* and designed forward RT-PCR primers with 3′ ends that terminate at sequence differences. After RT-PCR, sequencing was performed using a nested sequencing primer and we determined that our assays could unambiguously distinguish between mH2A2 and mH2A3 messages (Figure S1B).

With validated RT-PCR assays in hand, we determined the expression of mH2A forms in undifferentiated male (J1) and female (F121) ESCs. We found robust expression of H2afy1.2 and H2afy2 mRNA in these cells, but little or no H2afy1.1 mRNA (Figure S1C). In contrast, mouse embryonic fibroblasts (MEFs) showed robust expression of H2afy1.1 mRNA in addition to H2afy1.2 and H2afy2 mRNA (Figure S1C). Transcripts from the expressed pseudogene (*H2afy3*) were detected in all samples, albeit at low levels (Figure S1C). mRNA from the ubiquitously expressed gene encoding Gapdh was detected at uniform levels in all samples (Figure S1C).

We devised a strategy to use shRNA-mediated knock down of mH2A variants to create a series of male and female ESC lines that are doubly deficient for mH2A1.2/mH2A2 or mH2A1/mH2A2. A male J1 ESC line deficient for ESC-specific mH2As (mH2A1.2 and mH2A2) was identified and named J(kd)m1.2-m2. A second male ESC line with deficiency for both mH2A1 splice forms and mH2A2 was created and named J(kd)m1-m2. An analogous series of F121 female ESCs were produced and called F(kd)m1.2-m2 and F(kd)m1-m2. RT-PCR assays showed a robust depletion of both mH2A1.2 and mH2A2 in all analyzed knock down ESC lines (Figure S2A, C, E, and G) compared to double empty vector J(ev) and F(ev) and non-specific shRNA J(ns) and F(ns) control ESC lines. Western blots showed a corresponding depletion of mH2A1 protein in all knock down lines (Figure S2B, D, F, and H). We purchased three commercially available antibodies directed against mH2A2, but these did not have acceptable specificity as judged by both Western analysis and immunofluorescence (data not shown).

**Figure 1:**
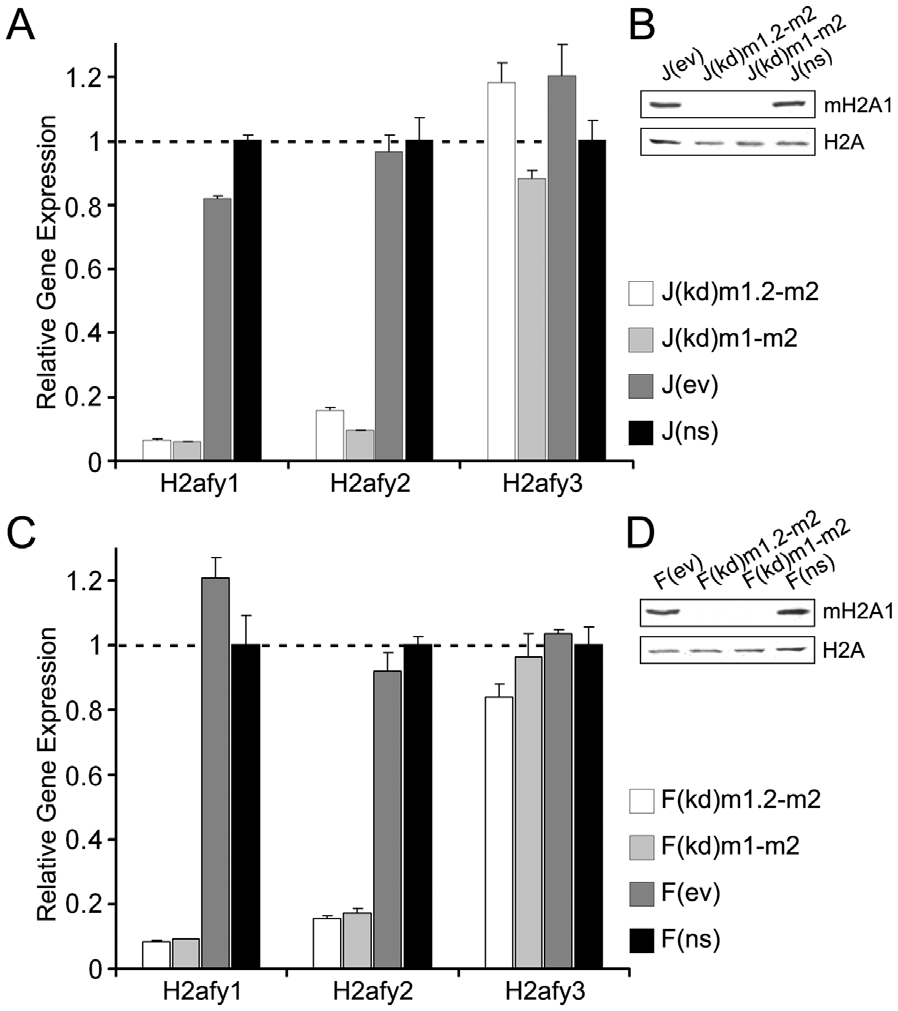
Stable knock down of macroH2A1 and macroH2A2 splice forms in male and female ES cells. (**A**) Stable shRNA-mediated knock down of ESC-specific mH2A variants (mH2A1.2 and mH2A2) and of all mH2A splice forms in male J1 ESCs (ESC lines J(kd)m1.2-m2 and J(kd)m1-m2, respectively), results in marked decrease of both mH2A1 (>90%) and mH2A2 (>85%) mRNAs, compared to empty vector (J(ev)) and non-specific (J(ns)) control ESC lines, assayed by quantitative RT-PCR. Expression levels of the pseudogene (*H2afy3*) remain uniform in all analyzed samples. Expression levels of individual mH2A variants are normalized to those of β-actin, using results from non-specific control ESC lines as the calibrator (set to a level of one). Results represent the average from three technical replicates and error bars represent standard deviation. (**B**) Western analysis demonstrates potent shRNA-mediated depletion of mH2A1 protein (>98%) in both knock down male ESC lines, compared to both control ESC lines. Protein levels of the canonical histone H2A are used as an unaffected loading control. (**C**) Quantitative RT-PCR assays in analogous series of female ESC lines show strong reduction of both H2afy1 (>90%) and H2afy2 (>80%) mRNAs in two knock down ESC lines (F(kd)m1.2-m2 and F(kd)m1-m2), compared to two control ESC lines (F(ev) and F(ns)). Expression levels of the pseudogene (*H2afy3*) remain unaffected. (**D**) Western blots show significant reduction of mH2A1 protein levels (>97%) in two female knock down ESC lines, compared to two control lines.

Quantitative RT-PCR analyses of selected knock down and control male and female ESC lines confirmed robust knock down of both mH2A1 (>90%) and mH2A2 (>85%) mRNAs in two male double knock down ESC lines (J(kd)m1.2-m2 and J(kd)m1-m2), while the expression of the pseudogene (*H2afy3*) was unaffected (Figure 1A). Significant depletion of H2afy mRNA in the knock down ESC lines resulted in >98% reduction of the mH2A1 protein (Figure 1B). Similar results were obtained for female knock down ESC lines (F(kd)m1.2-m2 and F(kd)m1-m2), in which >90% and >80% reduction of H2afy1 and H2afy2 mRNAs (respectively) could be observed by qRT-PCR (Figure 1C). This decrease of the message levels translated into >97% reduction of mH2A1 protein in both knock down ESC lines (Figure 1D).

### Proliferation and neuroectodermal differentiation of wild type and mH2A1/mH2A2-deficient ESCs

**Figure 2:**
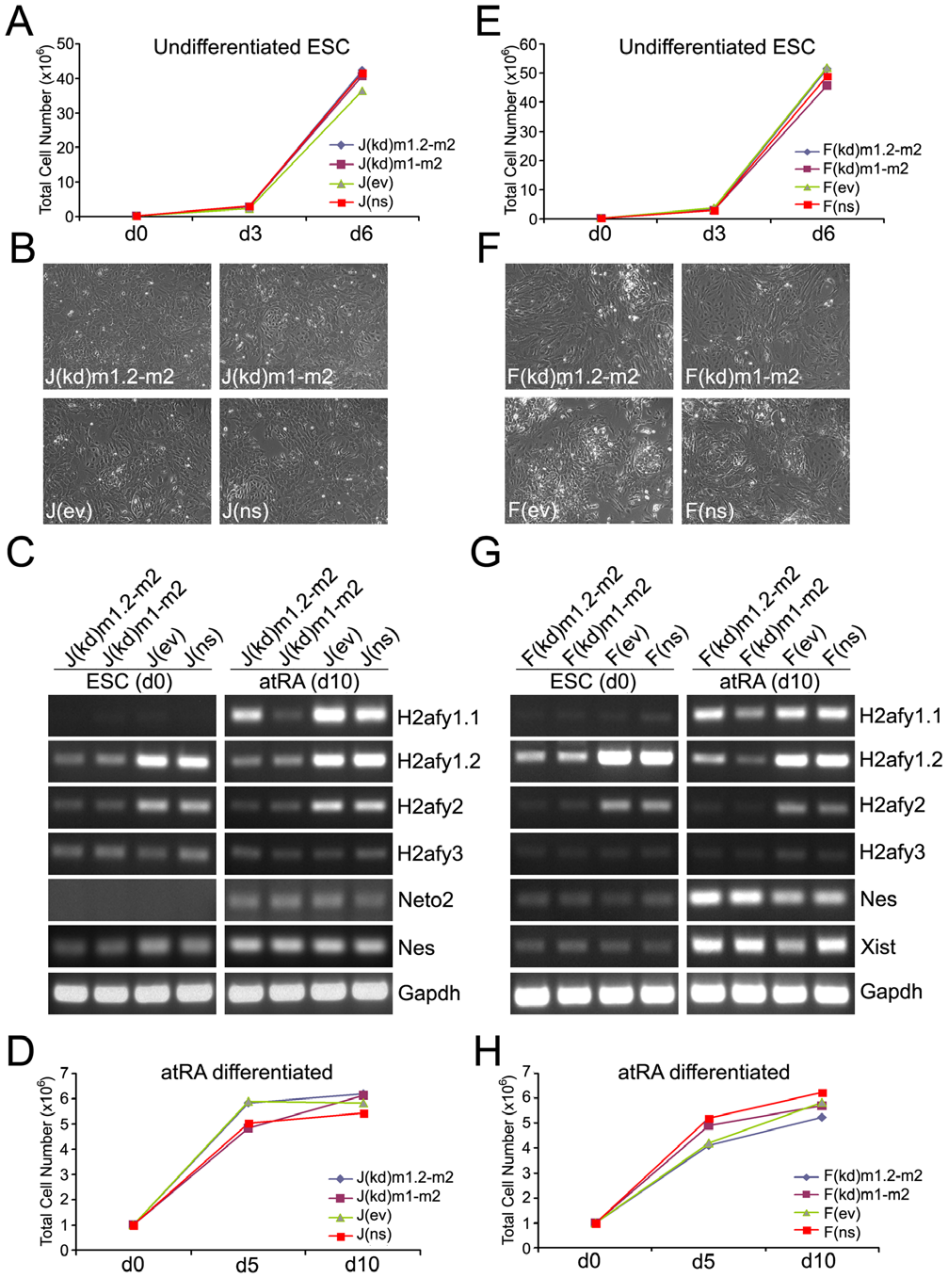
Neuroectodermal differentiation of male and female ESCs doubly-deficient for mH2A1 and mH2A2. (**A**) Double knock down and control male J1 ESCs exhibit nearly identical growth rates over the course of two passages in standard ESC medium. (**B**) Cellular morphology of male J1 cells after neuroectodermal differentiation in the presence of 100nM all-trans retinoic acid (atRA) at day 10 is shown. (**C**) Expression analysis of mH2A isoforms and ectoderm markers (Neto2 and Nes) in J1 wild type and mH2A double knock down ESCs in undifferentiated and atRA-differentiated states are shown. Note differentiation-induced expression of mH2A1.1 in all but the J(kd)m1-m2 sample. (**D**) Proliferation of knock down ESCs during 10 days of atRA differentiation is unaffected by mH2A1/mH2A2 shRNA-induced knock down. (**E–H**) Similar results are observed for female F121 knock down and control ESCs. Note that atRA differentiation strongly induces Xist in female cells.

Undifferentiated male and female double knock down ESCs exhibited highly similar proliferation rates during standard ESC culture and proliferation did not differ between wild type and control cell lines (Figure 2A, E). When exposed to all-trans retinoic acid (atRA), all cell lines exhibited changes in cellular morphology characteristic of neuroectodermal differentiation (Figure 2B, F). RT-PCR analysis demonstrated up-regulation of Neto2 and nestin (Nes) expression, independent markers of neuroectodermal lineage differentiation (Figure 2C, G). The differentiation procedure also strongly induced the production of mH2A1.1 mRNA in all cell lines, except J(kd)m1-m2 and F(kd)m1-m2, which contain an shRNA that targets all splice forms emanating from *H2afy*. atRA differentiation also induced robust Xist RNA expression in all female samples, suggesting that XCI can proceed in differentiating ESCs with greatly reduced mH2A1/mH2A2 content. Differentiated mH2A1/mH2A2-deficient cells exhibited growth rates comparable to control cell lines at days 5 and 10 after the initiation of differentiation (Figure 2D, H).

### X chromosome inactivation in ESCs deficient for mH2A1 and mH2A2

**Figure 3:**
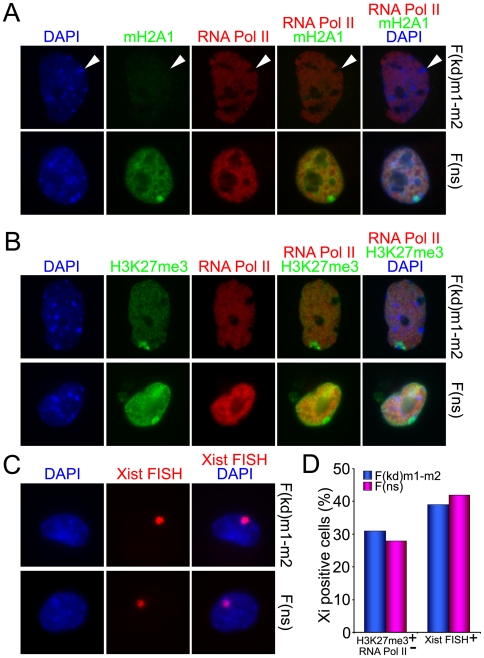
X chromosome inactivation in day 10 atRA-differentiated cells from female ESCs doubly-deficient for mH2A1 and mH2A2. (**A**) Nuclei from double knock down female line F(kd)m1-m2 exhibit a low mH2A1 signal and a perinuclear DAPI-dense region that is devoid of RNA Polymerase II, which marks the presumptive Xi (white arrowheads). Prominent macrochromatin bodies (MCBs) are readily observed in nuclei from a wild type control line F(ns) which overlap with regions devoid of RNA Pol II, indicating the presence of a single inactive X chromosome in each of these cells. (**B**) Both F(kd)m1-m2 and F(ns) samples show the presence of Xi, as judged by the presence of an Xi-specific histone modification signal (H3K27me3) over the RNA Pol II exclusion zone. (**C**) Xist RNA-FISH assays detect inactive X chromosomes in nuclei from mH2A1/mH2A2-deficient and control cell lines. (**D**) Similar numbers of cells with inactive X chromosomes were detected in female F(kd)m1-m2 and F(ns) control cell lines after atRA differentiation, as judged by two independent assays for X chromosome inactivation.

Excellent cell biology assays exist that can detect the presence of a single inactive X chromosome (Xi) in differentiated female cells. We therefore used a battery of cell biology approaches to determine the status of the XCI in the F(kd)m1-m2 ESC line, which contains a stable knock down of both mH2A1 and mH2A2. F(kd)m1-m2 female ESCs were induced to differentiate for 10 days in the presence of atRA, a differentiation procedure that is known to cause XCI in wild type female ESCs culminating in the formation of MCBs over the Xist RNA-FISH clouds [26]. We employed immunofluorescence protocols to detect the presence of Xi-specific epigenetic modifications (mH2A1 accumulation or H3K27me3 histone modification), combined with an assay for subnuclear sites devoid of interphase transcription as judged by the absence of RNA Polymerase II (RNA Pol II). As expected, an mH2A1 signal was virtually absent in nuclei from F(kd)m1-m2 samples (Figure 3A). However, presumptive inactive X chromosomes were detected as perinuclear DAPI-dense regions that were also devoid of RNA Pol II staining (Figure 3A). In contrast, prominent MCBs were detected over the RNA Pol II exclusion zones in nuclei from a wild type F(ns) control line (Figure 3A). Both mH2A1/mH2A2-deficient (F(kd)m1-m2) and wild type (F(ns)) lines exhibited H3K27me3 staining coincident with regions lacking an RNA Pol II signal (Figure 3B), corresponding to locations of Xi in interphase nuclei. In addition, Xist RNA-FISH demonstrated the presence of a single Xi located at peripheries of nuclei in both knock down and non-specific control lines (Figure 3C). Two hundred cells were counted for the latter two assays, and the number of cells exhibiting XCI was unperturbed by the knock down of mH2A1/mH2A2 (Figure 3D).

### Additional differentiation assays of mH2A1/mH2A2-deficient ESCs

**Figure 4:**
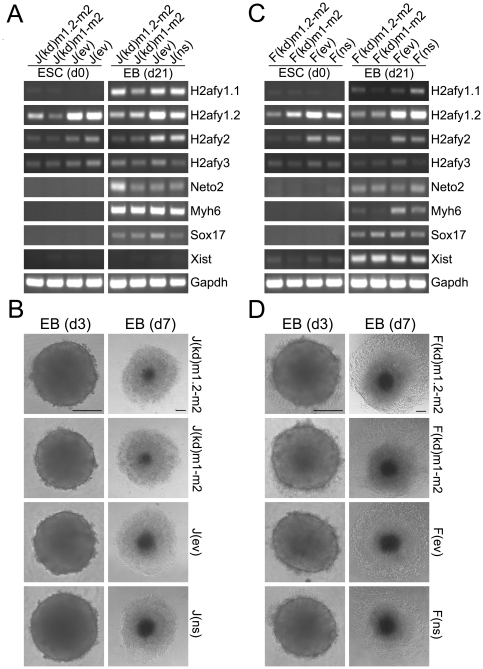
ESCs deficient for both mH2A1 and mH2A2 differentiate with similar kinetics into EBs containing cells of all three germ layers. (**A**) Differentiation of male J1 ESCs by EB formation induces up-regulation of mH2A1.1 (in all samples but J(kd)m1-m2), in addition to ectoderm (Neto2), mesoderm (Myh6), and endoderm (Sox17) markers. Knock down of mH2As was maintained through 21 days of EB differentiation. (**B**) EBs of uniform size were efficiently formed from 5,000 starting cells from all ESC lines at day 3 (d3). Further differentiation of EBs was detectable after 4 additional days of adherent growth on gelatinized dishes (d7). (**C**) Female knock down and control ESCs up-regulate Xist in day 21 EBs, in addition to markers of the three germ layers. (**D**) Female ESCs readily form EBs of uniform size (d3) that differentiate from the core outwards after plating on gelatin (d7). Scale bars = 200 µm.

The pluripotency of double knock down and control cell lines was tested *in vitro* by the formation of embryoid bodies (EBs). All cell lines readily formed EBs by random aggregation, and gene expression analyses confirmed the presence of markers for all three germ layers, ectoderm (Neto2), mesoderm (Myh6), and endoderm (Sox17) (Figure 4A, C). Female ESCs were slightly less efficient in up-regulating the mesoderm marker Myh6, while in male J1 ESCs this marker was strongly expressed in day 21 EBs. As expected, F121 transgenic knock down ESCs showed strong up-regulation of Xist expression at this EB stage, while the Xist expression in male ESC lines was virtually undetectable (Figure 4A, C). Robust knock down of mH2A1.2 and mH2A2 was maintained in day 21 EBs. The differentiation-induced up-regulation of mH2A1.1 was observed in day 21 EBs in male samples, except for the universal mH2A1/mH2A2 knock down line J(kd)m1-m2, as expected (Figure 4A). Female EBs showed a less efficient but detectable up-regulation of mH2A1.1 (Figure 4C). To further investigate the developmental potential of mH2A-deficient ESCs, synchronized EBs were created by initially aggregating a defined number of ESCs. Six EBs were formed for each cell line. All cell lines formed virtually identical EBs after three days. After adherence of EBs to a gelatinized substratum, differentiated halos spread from the compact core of EBs (Figure 4B, D). In all cases EBs exhibited uniform size and differentiation regardless of their knock down status. We conclude that *in vitro* differentiation into cells representative of all three germ layers occurs in ESCs depleted of mH2A1 and mH2A2.

We next wanted to determine the pluripotency of the mH2A-deficient ESCs *in vivo* by the formation of teratomas. Both knock down and control ESCs readily formed teratomas, and presumptive derivatives of all three germ layers could be observed on hematoxylin and eosin (H&E) stained sections (Figure S3A, C). RT-PCR analyses of mH2A variant expression in RNA extracted from these teratomas showed detectable mH2A transcripts in all teratomas. This is possibly a consequence of the inability to maintain selective conditions for the shRNA construct for the duration of the experiment, which could, in turn, lead to a silencing or loss of the shRNA transgene. Alternatively, contamination from the host NOD/SCID mouse during dissection and/or vascularization during teratoma formation could introduce host cells into teratoma samples. The second notion is supported by the finding that male ESCs that were introduced into female hosts yielded teratomas with detectable Xist expression, indicating the presence of RNA of host origin (Figure S3B). Efficient differentiation of F121 ESCs into teratomas was demonstrated by robust Xist expression, which occurred in female teratomas grown in male NOD/SCID mice (Figure S3D).

**Figure 5:**
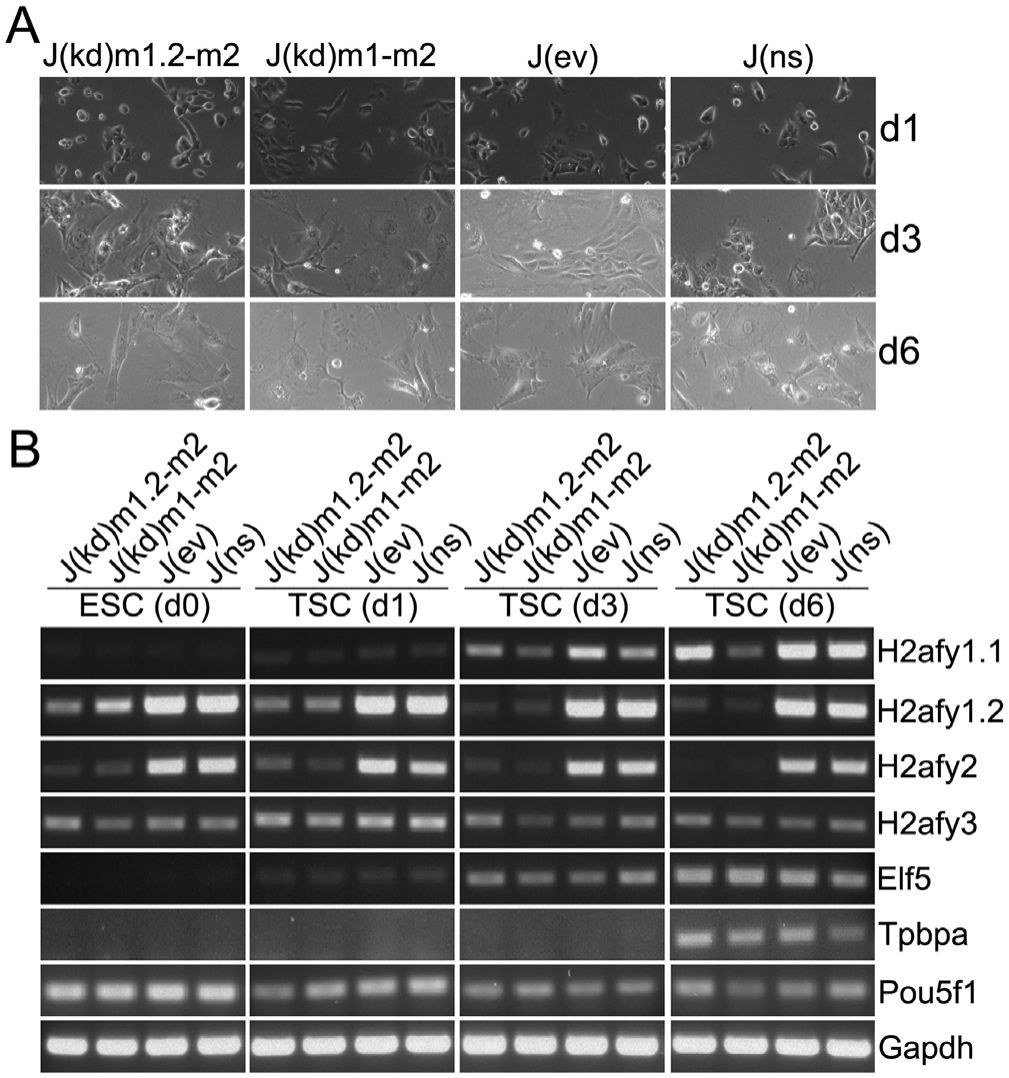
J1 ESCs deficient for mH2A1 and mH2A2 readily differentiate into trophoblast cells. (**A**) Phase images of cells after culture of ESCs in the absence of LIF under trophoblast stem cell (TSC) conditions (bFGF, heparin and 1 µM of 5-aza-dC). (**B**) RT-PCR assays show up-regulation of the early trophoblast marker Elf5 as early as day 1, while expression of the intermediate trophoblast marker Tpbpa is evident at day 6. Strong up-regulation of mH2A1.1 in all samples except J(kd)m1-m2 is also indicative of efficient differentiation.

Finally, we aimed to determine if mH2A1/mH2A2-deficient ESCs could differentiate *in vitro* into trophoblast cells. Trophoblast differentiation was performed as described by adding the DNA methylation inhibitor 5-aza-dC, bFGF, and heparin instead of LIF [27]. The procedure induced rapid and robust differentiation in all J1 transgenic lines as judged by phenotypic changes of the cells in the culture during the course of the experiment (Figure 5A). Combined puromycin and G418 selection maintained a robust knock down of targeted mH2As during the course of the experiment (Figure 5B). Transcription factor Elf5, a key regulator of trophoblast lineage commitment, was up-regulated as early as day 1. A later marker of trophoblast differentiation, Tpbpa was detected at day 6. The expression levels of the pluripotency marker Oct4 (Pou5f1) gradually decreased over the course of the experiment (Figure 5B). Trophoblast-directed differentiation of F121 knock down and control cell lines resulted in extensive cell death by day 3 after the induction (data not shown) for reasons unknown, thus precluding the analysis of mH2A function in the context of imprinted XCI.

### Imprints in ESCs deficient for mH2A1 and mH2A2

The preferential deposition of mH2A1 into the control regions of the inactive allele has been demonstrated for a subset of imprinted genes [21]. The F1 hybrid genetic background (*M.musculus/M.castaneus*) of the F121 ESC line allowed for allelic discrimination based on SNP analyses, and we detected informative SNPs in expressed regions of *Peg3* and *Dlk1* (Supplemental information, Materials and Methods). The presence of the expressed SNPs was confirmed by the direct sequencing of the PCR products using genomic DNA as a template (data not shown). RT-PCR results indicate that the *Peg3* locus is not imprinted in undifferentiated ESCs. Bi-allelic expression was observed in all of the analyzed ESC lines, as both nucleotides could be detected at the SNP position in chromatogram traces (Figure S4A). In contrast, *Dlk1* demonstrated a skewed allelic expression status, with only a minor contribution from the imprinted allele (Figure S4B). As expected, Dlk1 showed predominantly paternal expression, as the *M.castaneus* allele of paternal origin was the dominant one on the sequence traces. No difference in expression could be detected in knock down versus control ESC lines.

## Discussion

The scientific literature contains a number of prominent studies that show that mH2As associate with the Xi in both mice and humans [2], [3], [6], [7], [8], [9], [11], [12], [22]. However, both mice and humans harbor two genes encoding distinct mH2A histone variants [2], [3]. In addition, alternative splicing occurs for H2afy mRNAs in mice [4]. Redundancy has complicated the production of knock out mice that lack all macroH2As. The targeted single gene deletion of mH2A1 in mice results in a surprisingly mild phenotype, with animals that are viable and fertile, showing only subtle defects in glucose metabolism and lipid homeostasis [28], [29]. The fertility of male knock out mice suggests that the inactivation of sex chromosomes in XY bodies of developing sperm is not dependent on mH2A1 alone, even though localization of this histone variant to XY bodies has been reported [13]. Since mH2A1 knock out female mice are viable, XCI can clearly proceed in the absence of mH2A1 *in vivo*. In contrast, knock down of an mH2A2-like ortholog in zebrafish embryos resulted in severe and specific developmental defects [25]. It should be noted that zebrafish embryos express only mH2A2, even though the zebrafish genome contains genes for both mH2A subtypes. Consideration of the mouse and zebrafish data together suggested functional redundancy between mH2A1 and mH2A2 in mice. Based on these suggestive reports, we were keen to assess the functional significance of mH2As for XCI by using a genetic approach and we devised an RNAi-based strategy to stably disrupt mH2A1 and mH2A2 simultaneously in female mouse ESCs, an established model system for the investigation of XCI. To our surprise, we found that ESCs tolerated mH2A1/mH2A2 double deficiency well, and maintained their ability to undergo several types of differentiation. Strikingly, we found that female ESCs readily executed and maintained XCI even when levels of both mH2A1 and mH2A2 were drastically reduced. This finding strongly suggests that XCI can occur in mammals even with significantly reduced amounts of mH2A proteins.

Our results also suggest that heterochromatin, such as that present in the Xi, likely makes use of several epigenetic strategies that collude to collectively maintain its integrity. Consistent with this hypothesis, we note that reactivation of a silenced transgene located on Xi was observed in mH2A1-deficient cells, but only when both DNA methylation and histone acetylation were also perturbed [12]. Recently, occupancy of large chromatin domains by mH2A1 and repressive H3K27me3 was reported, but expressed genes were also identified that contained mH2A1 [24]. In addition, several reports describe a possible synergism between mH2A1 and DNA methylation for the maintenance of the heterochromatin [21], [30], [31]. Results presented here indicate that XCI is not greatly affected in differentiated ESCs that are dually-deficient for mH2A1 and mH2A2. Therefore it seems likely that multiple mechanisms that contribute to heterochromatin stability exist in mammalian cells, and that disruption of heterochromatin integrity requires the action of multiple epigenetic insults.

Macrohistones are apparently conserved in all vertebrates. Orthologs have been identified in fish and birds, which do not achieve dosage compensation by means of XCI [32], [33]. Even though macrohistones are restricted to vertebrate chromatin, proteins with macro domains have been found in other organisms, including bacteria and RNA viruses, where they are likely involved in replication [32], a finding that suggests macro-domain proteins may have spread to the common ancestors of vertebrates by horizontal gene transfer followed by an insertion into an H2A histone gene. To date, the only rigorous analysis of deletion of all macrohistones in a vertebrate animal has been conducted in zebrafish, which are not complicated by the presence of multiple isoforms during embryogenesis [25]. Interestingly, mH2A-deficient zebrafish exhibit profound developmental defects, suggesting that a genetic double knock out of *H2afy* and *H2afy2* in mice might exhibit a similar phenotype. If so, this will provide good evidence that the developing embryo is a far more sensitive crucible for the detection of developmental defects than cell culture-based ESC models. In addition, mH2As are found in vertebrates that do not have a female XX, male XY sex chromosome system, also suggesting that mH2A incorporation into the mammalian Xi is a relatively late and derived event in evolutionary terms.

## Materials and Methods

### ESC culture, RNA interference, and plasmid integrations

Male (J1) and female (F121) mouse embryonic stem cells (ESCs) were previously described [4]. ESC lines were propagated on monolayers of mitotically-inactivated MEF feeders obtained from Tg(DR4)1Jae/J E13.5 embryos. Undifferentiated ESCs were grown in the presence of 1000 U/ml LIF at 37°C under 5% CO_2_ in humidified air, as previously described [34]. Stable RNA interference was induced by electroporation of shRNA-harboring plasmid constructs targeting specific mH2A variants (H2afy1.2, Origene (SKU: TR312537); H2afy1 and H2afy2, Open Biosystems (SKUs: RMM4534-NM_012015 and RMM4534-NM_207000, respectively). Empty vector (no shRNA) and non-specific (“scrambled” shRNA) vector plasmids obtained from both companies were used as controls. shRNA constructs against H2afy1.2 and H2afy1 were subcloned into plasmids containing *neo^r^*, and these were introduced into ESC lines that were already deficient for H2afy2 (*puro^r^* ) in order to create double transgenic ESC lines that simultaneously target both mH2A variants (H2afy1 and H2afy2). Since H2afy transcripts undergo alternative splicing, we constructed H2afy2-deficient ESC lines that also target all mH2A1 splice forms, or specifically the mH2A1.2 splice form only (leaving mH2A1.1 intact). Double empty vector and double non-specific shRNA control ESC lines (*puro^r^* and *neo^r^* ) were also created using both J1 and F121 ESC lines. shRNA transgene integrations were performed by electroporation using Bio-Rad Gene Pulser, and transgenic ESC colonies were selected in 2 µg/ml of Puromycin (Sigma) and 500 µg/ml of G418 (Invitrogen). Established ESC lines were maintained under selection throughout all experimental procedures.

### RT-PCR, qRT-PCR, and Western blots

Semi-quantitative RT-PCR and quantitative real-time RT-PCR (qRT-PCR) assays were performed as previously described, with modifications [35]. qRT-PCR analyses were performed using iTaq Fast SYBR Green Supermix with ROX (Bio-Rad), following the manufacturer's recommended conditions. Data was analyzed using the comparative cycle threshold (Ct) method, as described [36]. Primers used in the study are listed in the Supplemental Information. For Western blots, 30 µg of total protein was resolved on 5-15% gradient polyacrylamide gels, transferred to Hybond PVDF membranes (GE Healthcare), and probed with an anti-mH2A1 antibody [30]. Detection was performed using an HRP-conjugated secondary antibody (Goat anti-rabbit IgG, Thermo Scientific) and ECL reagent (GE Healthcare) according to the manufacturer's protocol. Protein band intensities were quantified using Carestream MI software (Carestream Health Inc).

### Neuroectoderm-directed differentiation and growth curves

Neuroectoderm-directed differentiation of ESC lines was performed by supplementing the culture media with 100nM all-trans retionoic acid (atRA) (Sigma) in the absence of LIF. Total RNA was isolated from cell cultures 10 days after the initiation of differentiation and subjected to RT-PCR. Analyses of growth rates were performed for undifferentiated ESCs and over the course of 10 days of atRA differentiation. Proliferation of undifferentiated ESCs was determined over two consecutive passages, using a hemocytometer counting chamber with an improved Neubauer ruling. For the atRA-differentiated samples, cells were plated at low density on gelatinized 100mm cell culture plates (1×10^6^ cells per plate) in duplicate cultures, and the total cell number was determined at days 5 and 10 after the initiation of differentiation.

### Immunofluorescence and RNA-FISH

Immunofluorescence studies were performed essentially as described [35]. Female (F121) ESCs deficient for both mH2A1 and mH2A2 (F(kd)m1-m2) and non-specific control ESC line (F(ns)) were differentiated for 10 days in the presence of atRA as described above. Differentiated cells were collected by trypsinization and plated onto gelatinized glass coverslips placed in 6-well cell culture plates. Plates were centrifuged for 1 minute at 200xg to promote adherence and cultures were maintained in differentiation media for an additional 24 hours. Cells were extracted in cytoskeletal buffer, permeabilized, subjected to cytoskeletal buffer again, and then fixed in paraformaldehyde. Primary antibodies used in the study were as follows: anti-mH2A1 [30], anti-H3K27me3 and anti-RNA Polymerase II clone CTD4H8 (both from Millipore). Secondary antibodies were Alexa Fluor 488 donkey anti-rabbit and Alexa Fluor 594 donkey anti-mouse (Invitrogen). Nuclei were counterstained with DAPI and coverslips were mounted using Vectashield Mounting Media (Vector Laboratories). A total of 200 nuclei were scored for the co-localized presence of the DAPI dense region (Barr bodies), and the presence of MCBs or the Xi-specific histone modification (H3K27me3), and exclusion of RNA Polymerase II. Xist RNA-FISH probes were prepared from an Xist cDNA fragment encompassing most of exon 1 (Rasmussen Lab), by random octamer priming in the presence of 10 mM Cy3-dCTP (GE Healthcare). Detailed RNA-FISH procedures were previously reported [35].

### Embryoid body (EB) formation

EBs were generated from male and female knock down and control ESC lines as previously described [34]. Total RNA was extracted from adherent EBs at day 21 and the expression of germ layer-specific genes was assessed by semi-quantitative RT-PCR. Gene-specific intron-spanning primers are listed in Table S1. Synchronized EBs were initiated in Sumilon Cell-tight plates (Wako) by plating 5×10^3^ undifferentiated feeder-free ESCs into wells of a 96-well plate containing 200 µl of culture media without LIF. After 3 days, EBs were transferred to individual gelatinized wells (in order to allow for attachment and differentiation) in 24-well cell culture plates and propagated for an additional 4 days. Six EBs from each cell line were scored size and differentiation over the course of the experiment.

### Trophoblast-directed differentiation

Directed differentiation of male (J1) knock down and control ESC lines into cells of trophoblast lineage was performed as previously described [27]. Briefly, standard trophoblast stem cell (TSC) media (containing 70% of TSC media pre-conditioned on a monolayer of MEFs for 72 hours) was supplemented with 25ng/ml bFGF (Invitrogen) and 1 µg/ml heparin (Sigma) in the presence of 1 µM 5-aza, 2′-deoxycytidine. Media was replaced daily, and total RNA was isolated at days 1, 3, and 6 after the initiation of differentiation and expression of early and intermediate trophoblast-specific genes (*Elf5* and *Tpbpa*, respectively) was assessed by RT-PCR. Primer sequences are listed in Table S1.

### Teratoma formation assays

For the formation of teratomas, feeder-free cultures of all eight ESC lines were obtained through two rounds of panning, with subsequent culture under 2xLIF for one additional passage. ESCs were collected and resuspended in PBS at approximately 1×10^6^ cells per 100 µl. A total of 200 µl of cell suspension was injected subcutaneously into both flanks of NOD/SCID mice. Female ESCs were injected into male mice only, while J1 ESCs were injected into mice of both sexes (mH2A1.2+mH2A2 knockdown and empty vector control ESC lines into male mice, and mH2A1+mH2A2 knockdown and non-specific control ESC lines into female mice). Three weeks after the injection, mice were sacrificed and teratomas were harvested, weighed, and dissected into halves. One half was submerged into 500 µl of RNAlater stabilization reagent (Qiagen) for subsequent RNA isolation, and the other half of each isolated teratoma was fixed in 4% PFA overnight at 4°C with agitation. Fixed teratomas were embedded in paraffin and processed for hematoxylin and eosin staining. Tissue samples for RNA extraction were disrupted with a Tissuemiser homogenizer (Fisher Scientific), and further homogenized using QIAshredder (Qiagen) before purification on RNA columns (Qiagen) following the manufacturer's protocol.

### Expression analyses of imprinted genes

The female ESC line F121 was derived from a blastocyst obtained from cross between females of *Mus musculus* background with males of *Mus castaneus* background [4]. The mouse genome informatics (MGI) database (www.informatics.jax.org) was used to obtain list of all SNPs between these two mouse strains. Imprinted genes for which the deposition of mH2A1 into control regions of inactive allele was previously shown [21] were screened for the presence of expressed SNPs (i.e. SNPs located in the exons retained in mature mRNAs). Two genes (*Peg3* and *Dlk1*) were found to have informative SNPs (unique SNP IDs were rs50075878 and rs50424874, respectively). The imprinting status of *Peg3* and *Dlk1* was assessed by direct sequencing of RT-PCR product using intron-spanning primers flanking the SNPs. RT-PCR products from both double knockdown and wild type control ESC lines for each of the two analyzed genes were sequenced using the nested primers. Sequences were analyzed by Sequence Scanner v1.0 software (Applied Biosystems). Primers used in this analysis are listed in Table S1.

## Supporting Information

Figure S1 **Characterization of murine macrohistone isoform expression.** (**A**) Genomic organization of the *H2afy* and *H2afy2* loci on chromosomes 13 and 10, respectively. (**B**) Blast comparison of expressed sequences from gene *H2afy2* and the similar expressed pseudogene *H2afy3* (numbers indicate nucleotide position of the H2afy2 mRNA relative to the transcription start site). Forward RT-PCR primers are designed so that 3′ ends terminate at the base that differs between H2afy2 and H2afy3 mRNAs (H2afy2, blue arrow; H2afy3, red arrow). Following amplification using a reverse primer that is located in the identity region (green arrow), RT-PCR products were directly sequenced using a nested primer (gray arrow). Sequence chromatograms for the RT-PCR assays show specificity for the primers used in the study, which distinguish between H2afy2 and H2afy3 (sequence from blast comparison showed for reference, gray box). A reverse primer used in qRT-PCR analyses for both H2afy2 and H2afy3 mRNAs is also shown (yellow arrow). (**C**) RT-PCR expression analysis of all three macrohistone subtypes and the expressed *H2Afy3* pseudogene in male ESC line J1, female ESC line F121, and in mouse embryonic fibroblasts (MEF). RT-PCR signal of the ubiquitously expressed Gapdh was used as a sample loading control.
(TIF)

Figure S2 **Stable shRNA-mediated knock down of mH2A1 and mH2A2 splice forms in male and female ESCs.** RT-PCR and Western blot results are shown for double knock down (kd), empty vector (ev), and non-specific shRNA (ns) control male (J1) (**A–D**) and female (F121) ESC lines (**E–H**).Three independent knock down ESC lines were examined for each double knock down combination. Stable shRNA-mediated knock down of mH2A variants (mH2A1.2 and mH2A2) in male J1 ESCs (J(kd)m1.2-m2) assayed by RT-PCR (**A**), and Western analysis, using H2A as an unaffected loading control (**B**). Stable shRNA-mediated knock down of all splice forms of mH2A1 and mH2A2 variants in male J1 ESCs (J(kd)m1-m2) assayed by RT-PCR (**C**), and Western analysis (**D**). Stable shRNA-mediated knock down of mH2A variants (mH2A1.2 and mH2A2) in female F121 ESCs (F(kd)m1.2-m2) assayed by RT-PCR (**E**), and Western analysis (**F**). Stable shRNA-mediated knock down of all splice forms of mH2A1 and mH2A2 mH2A variants in female F121 ESCs (F(kd)m1-m2) assayed by RT-PCR (**G**), and Western analysis (**H**). Note that the H2afy1.1 isoform is not expressed in undifferentiated ESCs and thus the results are not shown. Expression levels for the expressed pseudogene (*H2afy3*) are shown for all analyzed samples.
(TIF)

Figure S3 **MacroH2A-deficient ESCs retain pluripotency and differentiate into all three germ layers in teratomas.** (**A**) Presumptive ectoderm, mesoderm, and endoderm cells are found in teratomas obtained from knock down (J(kd)m1.2-m2 and J(kd)m1-m2) and control (J(ev) and J(ns)) J1 ESCs. (**B**) Knock down levels were reduced in J(kd)m1.2-m2 and J(kd)m1-m2 teratoma samples. Xist expression in J(kd)m1-m2 and J(ns) teratomas are indicative of contamination with female host cells from NOD/SCID mice, since male cells were introduced into female hosts. (**C**) Female knock down (F(kd)m1.2-m2 and F(kd)m1-m2) and control (F(ev) and F(ns)) ESCs form cells representative of all three germ layers in teratomas. (**D**) RT-PCR results showing efficient up-regulation of Xist in female samples.
(TIF)

Figure S4 **Allelic expression of imprinted genes is unaffected by mH2A depletion.** (**A**) The *Peg3* gene is not imprinted in female *M.musculus/M.castaneus* hybrid F1 ESCs and exhibits biallelic expression at the SNP position (represented by dual-colored rhombus). The sequence was produced by sequencing of RT-PCR products in regions containing expressed SNPs. (**B**) A maternal imprint persists for the *Dlk1* locus in mH2A1/mH2A2-deficient cells.
(TIF)

Table S1 **List of primer sequences.**
(PDF)

## References

[1] Pehrson JR, Fried VA (1992). MacroH2A, a core histone containing a large nonhistone region.. Science.

[2] Chadwick BP, Willard HF (2001). Histone H2A variants and the inactive X chromosome: identification of a second macroH2A variant.. Hum Mol Genet.

[3] Costanzi C, Pehrson JR (2001). MACROH2A2, a new member of the MARCOH2A core histone family.. J Biol Chem.

[4] Rasmussen TP, Huang T, Mastrangelo MA, Loring J, Panning B (1999). Messenger RNAs encoding mouse histone macroH2A1 isoforms are expressed at similar levels in male and female cells and result from alternative splicing.. Nucleic Acids Res.

[5] Kawai J, Shinagawa A, Shibata K, Yoshino M, Itoh M (2001). Functional annotation of a full-length mouse cDNA collection.. Nature.

[6] Costanzi C, Pehrson JR (1998). Histone macroH2A1 is concentrated in the inactive X chromosome of female mammals.. Nature.

[7] Mietton F, Sengupta AK, Molla A, Picchi G, Barral S (2009). Weak but uniform enrichment of the histone variant macroH2A1 along the inactive X chromosome.. Mol Cell Biol.

[8] Csankovszki G, Panning B, Bates B, Pehrson JR, Jaenisch R (1999). Conditional deletion of Xist disrupts histone macroH2A localization but not maintenance of X inactivation.. Nat Genet.

[9] Rasmussen TP, Wutz AP, Pehrson JR, Jaenisch RR (2001). Expression of Xist RNA is sufficient to initiate macrochromatin body formation.. Chromosoma.

[10] Mermoud JE, Costanzi C, Pehrson JR, Brockdorff N (1999). Histone macroH2A1.2 relocates to the inactive X chromosome after initiation and propagation of X-inactivation.. J Cell Biol.

[11] Costanzi C, Stein P, Worrad DM, Schultz RM, Pehrson JR (2000). Histone macroH2A1 is concentrated in the inactive X chromosome of female preimplantation mouse embryos.. Development.

[12] Hernandez-Munoz I, Lund AH, van der Stoop P, Boutsma E, Muijrers I (2005). Stable X chromosome inactivation involves the PRC1 Polycomb complex and requires histone MACROH2A1 and the CULLIN3/SPOP ubiquitin E3 ligase.. Proc Natl Acad Sci U S A.

[13] Hoyer-Fender S, Costanzi C, Pehrson JR (2000). Histone macroH2A1.2 is concentrated in the XY-body by the early pachytene stage of spermatogenesis.. Exp Cell Res.

[14] Zhang R, Poustovoitov MV, Ye X, Santos HA, Chen W (2005). Formation of MacroH2A-containing senescence-associated heterochromatin foci and senescence driven by ASF1a and HIRA.. Dev Cell.

[15] Agelopoulos M, Thanos D (2006). Epigenetic determination of a cell-specific gene expression program by ATF-2 and the histone variant macroH2A.. Embo J.

[16] Kwiatkowski DL, Thompson HW, Bloom DC (2009). The polycomb group protein Bmi1 binds to the herpes simplex virus 1 latent genome and maintains repressive histone marks during latency.. J Virol.

[17] Ouararhni K, Hadj-Slimane R, Ait-Si-Ali S, Robin P, Mietton F (2006). The histone variant mH2A1.1 interferes with transcription by down-regulating PARP-1 enzymatic activity.. Genes Dev.

[18] Doyen CM, An W, Angelov D, Bondarenko V, Mietton F (2006). Mechanism of polymerase II transcription repression by the histone variant macroH2A.. Mol Cell Biol.

[19] Angelov D, Molla A, Perche PY, Hans F, Cote J (2003). The histone variant macroH2A interferes with transcription factor binding and SWI/SNF nucleosome remodeling.. Mol Cell.

[20] Chang EY, Ferreira H, Somers J, Nusinow DA, Owen-Hughes T (2008). MacroH2A allows ATP-dependent chromatin remodeling by SWI/SNF and ACF complexes but specifically reduces recruitment of SWI/SNF.. Biochemistry.

[21] Choo JH, Kim JD, Chung JH, Stubbs L, Kim J (2006). Allele-specific deposition of macroH2A1 in imprinting control regions.. Hum Mol Genet.

[22] Changolkar LN, Pehrson JR (2006). macroH2A1 histone variants are depleted on active genes but concentrated on the inactive X chromosome.. Mol Cell Biol.

[23] Changolkar LN, Singh G, Cui K, Berletch JB, Zhao K (2010). Genome-Wide Distribution of MacroH2A1 Histone Variants in Mouse Liver Chromatin..

[24] Gamble MJ, Frizzell KM, Yang C, Krishnakumar R, Kraus WL (2010). The histone variant macroH2A1 marks repressed autosomal chromatin, but protects a subset of its target genes from silencing.. Genes Dev.

[25] Buschbeck M, Uribesalgo I, Wibowo I, Rue P, Martin D (2009). The histone variant macroH2A is an epigenetic regulator of key developmental genes.. Nat Struct Mol Biol.

[26] Rasmussen TP, Mastrangelo MA, Eden A, Pehrson JR, Jaenisch R (2000). Dynamic relocalization of histone MacroH2A1 from centrosomes to inactive X chromosomes during X inactivation.. J Cell Biol.

[27] Ng RK, Dean W, Dawson C, Lucifero D, Madeja Z (2008). Epigenetic restriction of embryonic cell lineage fate by methylation of Elf5.. Nat Cell Biol.

[28] Changolkar LN, Costanzi C, Leu NA, Chen D, McLaughlin KJ (2007). Developmental changes in histone macroH2A1-mediated gene regulation.. Mol Cell Biol.

[29] Boulard M, Storck S, Cong R, Pinto R, Delage H (2010). Histone variant macroH2A1 deletion in mice causes female-specific steatosis.. Epigenetics Chromatin.

[30] Ma Y, Jacobs SB, Jackson-Grusby L, Mastrangelo MA, Torres-Betancourt JA (2005). DNA CpG hypomethylation induces heterochromatin reorganization involving the histone variant macroH2A.. J Cell Sci.

[31] Barzily-Rokni M, Friedman N, Ron-Bigger S, Isaac S, Michlin D (2011). Synergism between DNA methylation and macroH2A1 occupancy in epigenetic silencing of the tumor suppressor gene p16(CDKN2A).. Nucleic Acids Res.

[32] Pehrson JR, Fuji RN (1998). Evolutionary conservation of histone macroH2A subtypes and domains.. Nucleic Acids Res.

[33] Araya I, Nardocci G, Morales J, Vera M, Molina A (2010). MacroH2A subtypes contribute antagonistically to the transcriptional regulation of the ribosomal cistron during seasonal acclimatization of the carp fish.. Epigenetics Chromatin.

[34] Ambrosi DJ, Tanasijevic B, Kaur A, Obergfell C, O'Neill RJ (2007). Genome-wide reprogramming in hybrids of somatic cells and embryonic stem cells.. Stem Cells.

[35] Tanasijevic B, Dai B, Ezashi T, Livingston K, Roberts RM (2009). Progressive accumulation of epigenetic heterogeneity during human ES cell culture.. Epigenetics.

[36] Livak KJ, Schmittgen TD (2001). Analysis of relative gene expression data using real-time quantitative PCR and the 2(-Delta Delta C(T)) Method.. Methods.

