# Supplementary figures and images for: X Chromosome Inactivation and Differentiation Occur Readily in ES Cells Doubly-Deficient for MacroH2A1 and MacroH2A2
Source: PLoS One. 2011 Jun 30;6(6):e21512. doi: 10.1371/journal.pone.0021512 (PMC3127949; doi:10.1371/journal.pone.0021512)

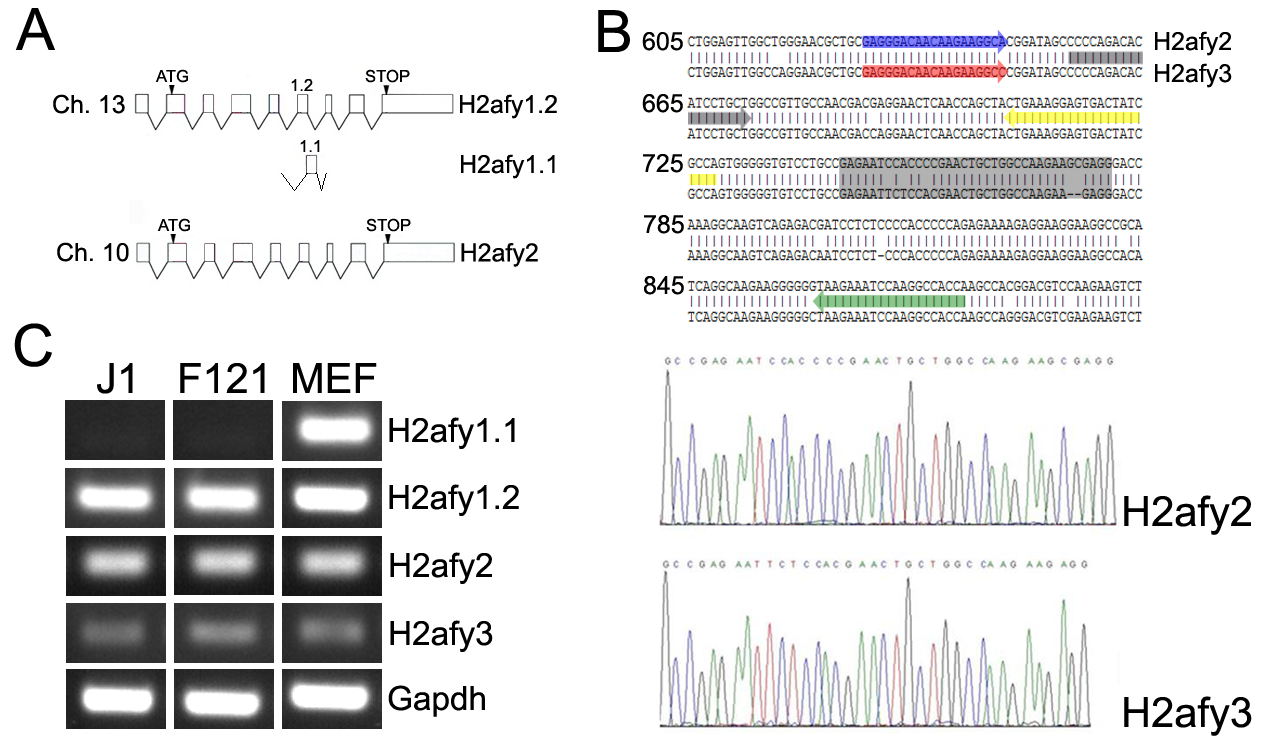

Supplement: Figure S1 — Characterization of murine macrohistone isoform expression. (A) Genomic organization of the H2afy and H2afy2 loci on chromosomes 13 and 10, respectively. (B) Blast comparison of expressed sequences from gene H2afy2 and the similar expressed pseudogene H2afy3 (numbers indicate nucleotide position of the H2afy2 mRNA relative to the transcription start site). Forward RT-PCR primers are designed so that 3′ ends terminate at the base that differs between H2afy2 and H2afy3 mRNAs (H2afy2, blue arrow; H2afy3, red arrow). Following amplification using a reverse primer that is located in the identity region (green arrow), RT-PCR products were directly sequenced using a nested primer (gray arrow). Sequence chromatograms for the RT-PCR assays show specificity for the primers used in the study, which distinguish between H2afy2 and H2afy3 (sequence from blast comparison showed for reference, gray box). A reverse primer used in qRT-PCR analyses for both H2afy2 and H2afy3 mRNAs is also shown (yellow arrow). (C) RT-PCR expression analysis of all three macrohistone subtypes and the expressed H2Afy3 pseudogene in male ESC line J1, female ESC line F121, and in mouse embryonic fibroblasts (MEF). RT-PCR signal of the ubiquitously expressed Gapdh was used as a sample loading control. (TIF) [file pone.0021512.s001.tif]

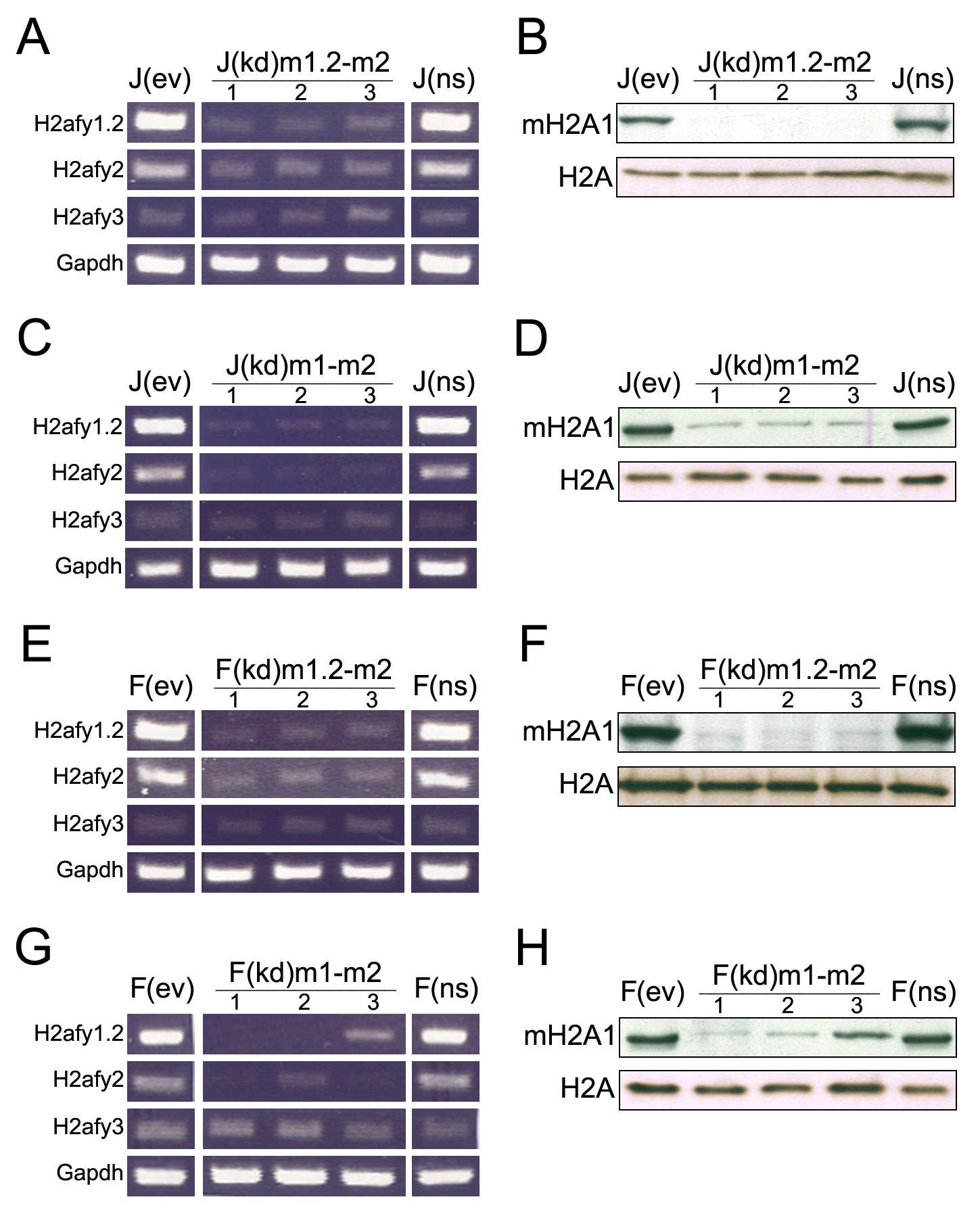

Supplement: Figure S2 — Stable shRNA-mediated knock down of mH2A1 and mH2A2 splice forms in male and female ESCs. RT-PCR and Western blot results are shown for double knock down (kd), empty vector (ev), and non-specific shRNA (ns) control male (J1) (A–D) and female (F121) ESC lines (E–H).Three independent knock down ESC lines were examined for each double knock down combination. Stable shRNA-mediated knock down of mH2A variants (mH2A1.2 and mH2A2) in male J1 ESCs (J(kd)m1.2-m2) assayed by RT-PCR (A), and Western analysis, using H2A as an unaffected loading control (B). Stable shRNA-mediated knock down of all splice forms of mH2A1 and mH2A2 variants in male J1 ESCs (J(kd)m1-m2) assayed by RT-PCR (C), and Western analysis (D). Stable shRNA-mediated knock down of mH2A variants (mH2A1.2 and mH2A2) in female F121 ESCs (F(kd)m1.2-m2) assayed by RT-PCR (E), and Western analysis (F). Stable shRNA-mediated knock down of all splice forms of mH2A1 and mH2A2 mH2A variants in female F121 ESCs (F(kd)m1-m2) assayed by RT-PCR (G), and Western analysis (H). Note that the H2afy1.1 isoform is not expressed in undifferentiated ESCs and thus the results are not shown. Expression levels for the expressed pseudogene (H2afy3) are shown for all analyzed samples. (TIF) [file pone.0021512.s002.tif]

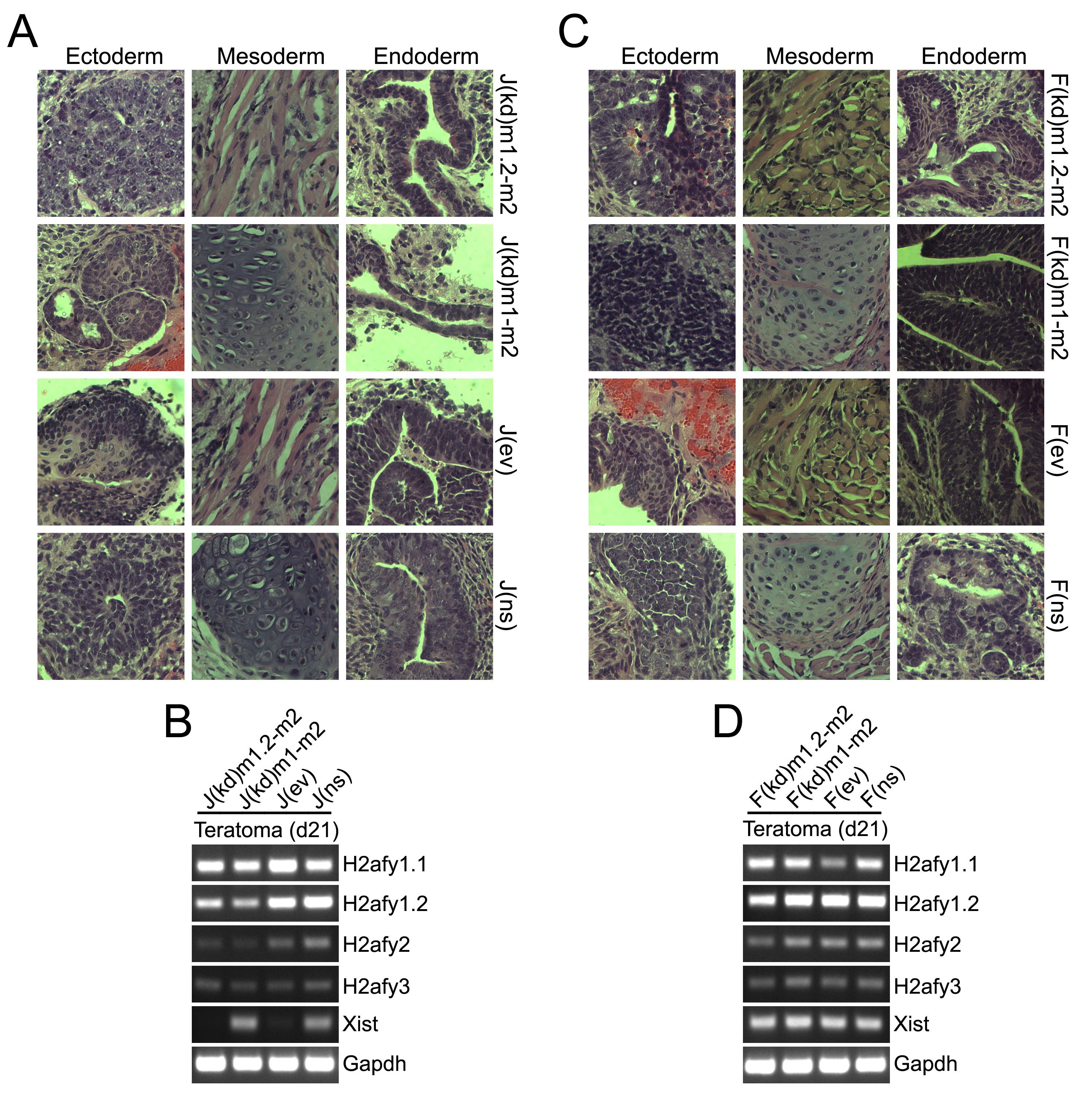

Supplement: Figure S3 — MacroH2A-deficient ESCs retain pluripotency and differentiate into all three germ layers in teratomas. (A) Presumptive ectoderm, mesoderm, and endoderm cells are found in teratomas obtained from knock down (J(kd)m1.2-m2 and J(kd)m1-m2) and control (J(ev) and J(ns)) J1 ESCs. (B) Knock down levels were reduced in J(kd)m1.2-m2 and J(kd)m1-m2 teratoma samples. Xist expression in J(kd)m1-m2 and J(ns) teratomas are indicative of contamination with female host cells from NOD/SCID mice, since male cells were introduced into female hosts. (C) Female knock down (F(kd)m1.2-m2 and F(kd)m1-m2) and control (F(ev) and F(ns)) ESCs form cells representative of all three germ layers in teratomas. (D) RT-PCR results showing efficient up-regulation of Xist in female samples. (TIF) [file pone.0021512.s003.tif]

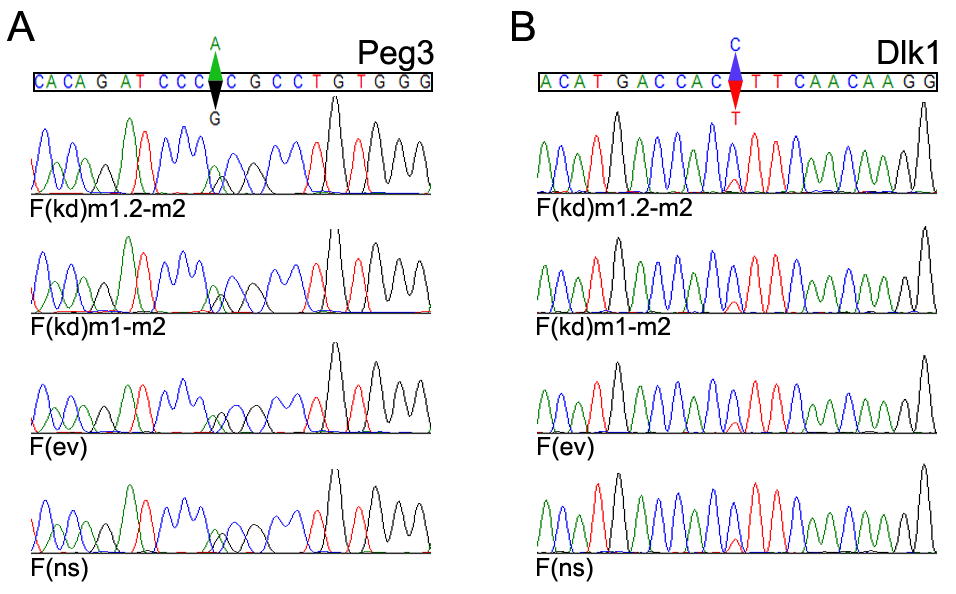

Supplement: Figure S4 — Allelic expression of imprinted genes is unaffected by mH2A depletion. (A) The Peg3 gene is not imprinted in female M.musculus/M.castaneus hybrid F1 ESCs and exhibits biallelic expression at the SNP position (represented by dual-colored rhombus). The sequence was produced by sequencing of RT-PCR products in regions containing expressed SNPs. (B) A maternal imprint persists for the Dlk1 locus in mH2A1/mH2A2-deficient cells. (TIF) [file pone.0021512.s004.tif]
