# Supplementary material for: X Chromosome Inactivation and Differentiation Occur Readily in ES Cells Doubly-Deficient for MacroH2A1 and MacroH2A2
Source: PLoS One. 2011 Jun 30;6(6):e21512. doi: 10.1371/journal.pone.0021512 (PMC3127949; doi:10.1371/journal.pone.0021512)
Supplement: Table S1 — List of primer sequences. (PDF) [file pone.0021512.s005.pdf]

Tanasijevic B, Rasmussen TP – Supplemental information

Table S1. Complete list of primers used in the study

Primer Name	Primer Sequence (5'-3')
**H2afy1.1_Fwd**	CTTTGAGGTGGAGGCCATAA
**H2afy1.2_Fwd**	CAGTGATGCTGTCGTTCACC
**H2afy1_Rev**	GAGCCAAGCAGTTTTTCACC
**H2afy2_Fwd**	GAGGGACAACAAGAAGGCA
**H2afy3_Fwd**	GAGGGACAACAAGAAGGCC
**H2afy2/3_Rev**	TGGTGGCCTTGGATTTCTTA
**qH2afy2/3_Rev**	TGGCGATAGTCACTCCTTTCAG
**H2afy2/3_Seq**	CCCCAGACACATCCTGCT
**Gapdh_Fwd**	AACTTTGGCATTGTGGAAGG
**Gapdh_Rev**	ACACATTGGGGGTAGGAACA
**qH2afy1_Fwd**	TCGCCTTCCCATCCATTG
**qH2afy1_Rev**	AGCTGGAGATGGCCTTCAGA
**qActb_Fwd**	GTGAAAAGATGACCCAGATCA
**qActb_Rev**	CACAGCCTGGATGGCTACGT
**Neto2_Fwd**	GCAGTTGATTGCATATGGACA
**Neto2_Rev**	TTCATTTGAGTGTTCCATTTGG
**Nes_Fwd**	AGCAGGAGAAGCAGGGTCTA
**Nes_Rev**	CTGGGAACCTTCTTCCAGGTG
**Myh6_Fwd**	ATGTTAAGGCCAAGGTCGTG
**Myh6_Rev**	ACGCAGAAGAGGCCTGAGTA
**Sox17_Fwd**	GGAGGGTCACCACTGCTTTA
**Sox17_Rev**	AGATGTCTGGAGGTGCTGCT
**Xist_Fwd**	GTGCTCCTGCCTCAAGAAGA
**Xist_Rev**	TTTTTCTCCATGTGGCCTGT
**Oct4_Fwd**	CCAATGCCGTGAAGTTGGA
**Oct4_Rev**	GCTCCTTCTGCAGGGCTTT
**Elf5_Fwd**	GGACTCCGTAACCCATAGCA
**Elf5_Rev**	TACTGGTCGCAGCAGAATTG
**Tpbpa_Fwd**	TGAAGAGCTGAACCACTGGA
**Tpbpa_Rev**	CTTGCAGTTCAGCATCCAAC
**Peg3_Fwd**	ACATGACACAAGGCCACTCA
**Peg3_Rev**	GTTCAGCTCCCTTGCTCTTC
**Peg3_Seq**	CGAGCCACATCCTTGATGA
**Dlk1_Fwd**	CACCTGGGTTCTCTGGAAAG
**Dlk1_Rev**	CTCATCACCAGCCTCCTTGT
**Dlk1-Seq**	CTCAACAAGTGCGAAACCTG
